# Extracellular nicotinamide phosphoribosyltransferase (eNAMPT) is a novel marker for patients with BRAF-mutated metastatic melanoma

**DOI:** 10.18632/oncotarget.24871

**Published:** 2018-04-10

**Authors:** Valentina Audrito, Antonella Managò, Federica Zamporlini, Eliana Rulli, Federica Gaudino, Gabriele Madonna, Stefania D'Atri, Gian Carlo Antonini Cappellini, Paolo Antonio Ascierto, Daniela Massi, Nadia Raffaelli, Mario Mandalà, Silvia Deaglio

**Affiliations:** ^1^ Department of Medical Sciences, University of Turin, Turin, Italy; ^2^ Italian Institute for Genomic Medicine, Turin, Italy; ^3^ Department of Agricultural, Food and Environmental Sciences, Polytechnic University of Marche, Ancona, Italy; ^4^ Statistics Unit Methodology for Clinical research Laboratory, Oncology Department, IRCCS, Istituto di Ricerche Farmacologiche Mario Negri, Milan, Italy; ^5^ Melanoma, Cancer Immunotherapy and Innovative Therapies O.U., Istituto Nazionale Tumori IRCCS Fondazione “G. Pascale”, Napoli, Italy; ^6^ Laboratory of Molecular Oncology, Istituto Dermopatico dell'Immacolata-IRCCS, Rome, Italy; ^7^ Department of Oncology and Dermatological Oncology, Istituto Dermopatico dell'Immacolata-IRCCS, Rome, Italy; ^8^ Division of Pathological Anatomy, Department of Surgery and Translational Medicine, University of Florence, Florence, Italy; ^9^ Unit of Medical Oncology, Department of Oncology and Hematology, Papa Giovanni XXIII Hospital, Bergamo, Italy

**Keywords:** metastatic melanoma, tumor marker, prognosis, resistance to therapy, NAMPT

## Abstract

Metastatic melanoma carrying BRAF mutations represent a still unmet medical need as success of BRAF inhibitors is limited by development of resistance. Nicotinamide phosphoribosyltransferase (NAMPT) is a key enzyme in NAD biosynthesis. An extracellular form (eNAMPT) possesses cytokine-like functions and is up-regulated in inflammatory disorders, including cancer. Here we show that eNAMPT is actively released in culture supernatants of melanoma cell lines. Furthermore, cells that become resistant to BRAF inhibitors (BiR) show a significant increase of eNAMPT levels. Plasma from mice xenografted with BiR cell lines contain higher eNAMPT levels compared to tumor-free animals. Consistently, eNAMPT levels are elevated in 113 patients with BRAF-mutated metastatic melanoma compared to 50 with localized disease or to 38 healthy donors, showing a direct correlation with markers of tumor burden, such as LDH, or aggressive disease (such as PD-L1). eNAMPT concentrations decrease in response to therapy with BRAF/MEK inhibitors, but increase again at progression, as inferred from the serial analysis of 50 patients. Lastly, high eNAMPT levels correlate with a significantly shorter overall survival.

Our findings suggest that eNAMPT is a novel marker of tumor burden and response to therapy in patients with metastatic melanoma carrying BRAF mutations.

## INTRODUCTION

NAD is a vital molecule in all organisms and a major component of both energy and signal transduction processes [1, 2]. NAD can be synthesized from different routes, including a *de novo* pathway starting from tryptophan and various pathways that salvage the three forms of vitamin B3, namely nicotinamide, nicotinic acid and nicotinamide riboside (NR). Nicotinamide, which is released by NAD-metabolizing enzymes, is the major source to maintain NAD levels, linking substrate and product in a functional loop [3–6]. Nicotinamide is recycled back to NAD via a two-step pathway involving nicotinamide conversion to NMN, and NMN adenylation to NAD. The enzyme nicotinamide phosphoribosyltransferase (NAMPT) catalyzes the first and rate-limiting reaction of the pathway [7, 8]. Beside this canonical intracellular activity, NAMPT was discovered to be present in the extracellular milieu where it exerts cytokine/adipokine-like actions [eNAMPT, aka pre-B cell colony enhancing factor (PBEF) or Visfatin] [9]. Elevated eNAMPT levels are typical of acute and chronic inflammatory conditions [8, 10], metabolic disorders [11–14], and cancer [15, 16]. Even if the mechanisms underlying eNAMPT secretion remain unknown, there seems to be a direct correlation with intracellular (i)NAMPT concentration [8, 17, 18].

We recently studied eNAMPT functions in the plasma of patients with chronic lymphocytic leukemia (CLL), a disease where tumor-host interactions and local inflammation are critical in regulating disease progression. Our results indicate that eNAMPT levels correlate with disease burden and that eNAMPT creates favorable conditions for tumor growth, by contributing to the development of a population of type 2 macrophages [19].

The mechanisms of action of eNAMPT remain unclear, even if the enzymatic activity appears dispensable. The group of Garcia recently proposed that eNAMPT may bind toll-like receptor 4 (TLR4), activating its signaling pathway, at least in a model of lung endothelial cell injury [20]. Elevated eNAMPT levels were also described in supernatants from melanoma cell cultures, where both autocrine and paracrine functions were hypothesized [21].

Our recent data indicate that NAMPT becomes the master regulator of NAD synthesis in BRAF-mutated melanoma cells that acquire resistance to BRAF inhibitors (BRAFi) [22]. Consistently, these cells are uniquely sensitive to NAMPT inhibitors, both *in vitro* and *in vivo*.

Starting from these observations, we measured eNAMPT levels in melanoma cell lines and in a large cohort of patients with BRAF-mutated metastatic melanomas, before and after therapy, suggesting that eNAMPT is a novel disease marker in BRAF-mutated melanoma patients.

## RESULTS

### eNAMPT is present in melanoma cell line supernatants and in melanoma xenografts, increasing upon acquisition of resistance to BRAFi

**Figure 1:**
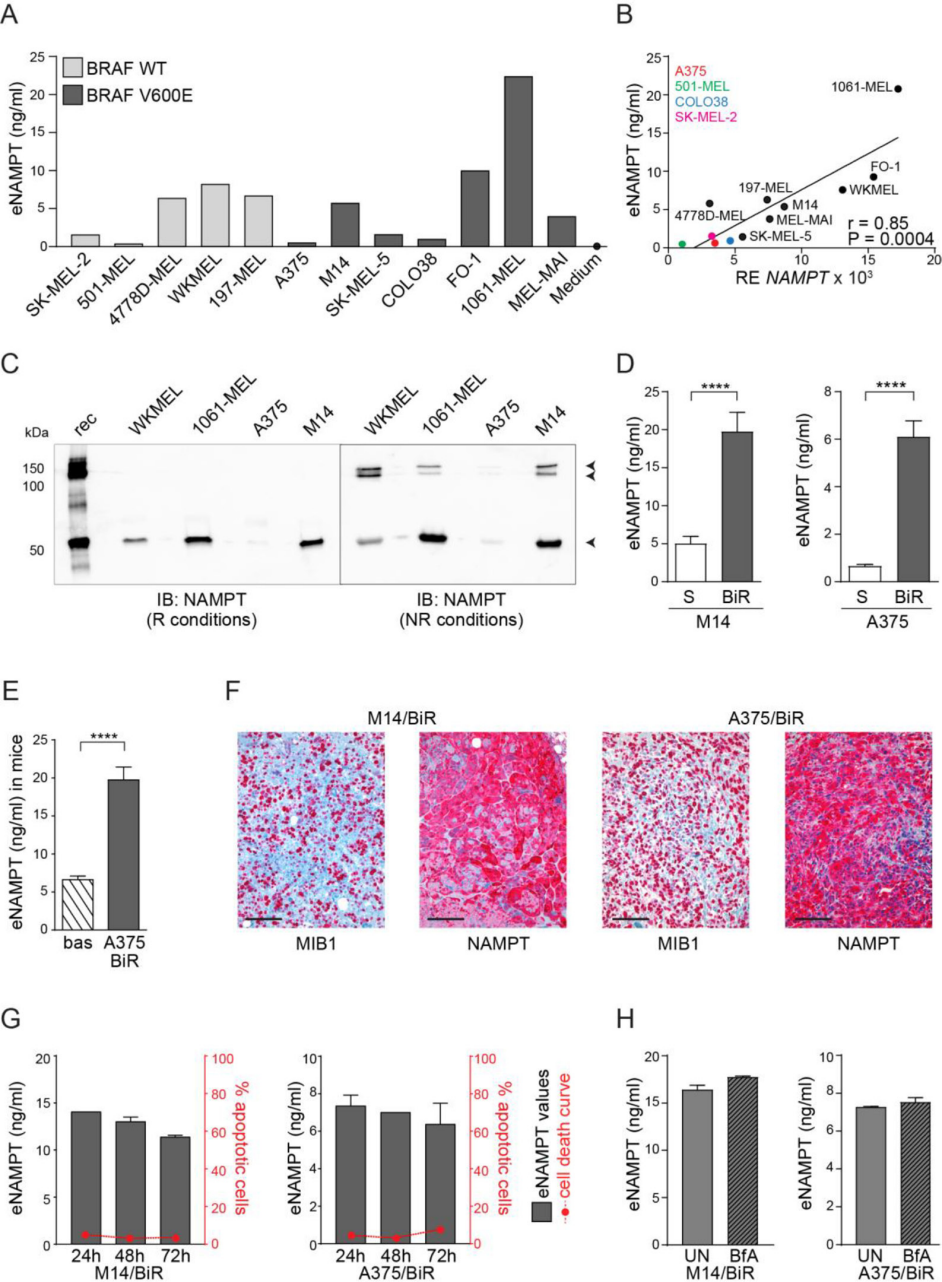
Melanoma cells release high eNAMPT levels (**A**) eNAMPT concentrations (ng/ml) measured with a quantitative ELISA assay in the supernatant (SN) of 5 BRAF-wt and 7 BRAF-mutated melanoma cell lines. Medium corresponds to RPMI 10% FCS. (**B**) Regression line showing a positive correlation between *NAMPT* mRNA levels (x-axis) and eNAMPT (y-axis) detected in the same 12 melanoma cell lines. Pearson coefficient (r) and the corresponding *P* value are noted. (**C**) The presence of eNAMPT was confirmed by western blot performed on 10× concentrated culture supernatants from WKMEL, 1061-MEL, M14 and A375 cell lines in reducing and not-reducing conditions. Rec (recombinant NAMPT) was loaded as control. (**D–E**) ELISA assay showing eNAMPT concentrations in SN from M14/S and /BiR (*n* = 10) and A375/S and /BiR (*n* = 10) (D) or in plasma from NSG mice xenografted by subcutaneous injection of A375/BiR cell lines (*n* = 8). Bas: eNAMPT levels in plasma collected before tumor xenotransplantation, A375/BiR: eNAMPT levels in plasma collected after tumor masses reached 1 mm^3^ (E). (**F**) Representative images of MIB1/NAMPT staining in tumor sections derived from NSG mice xenografted with BiR cells. Original magnification 20×. Scale bar = 100 μm. (**G**) Graphs showing eNAMPT values in the SN of M14/BiR and A375/BiR measured at 24, 48 and 72 hours (black left y axis, *n* = 3) and concomitant % of apoptotic cells (AnnexinV^+^/propidium iodide^+^) in the same cells for the time indicated (red right y axis, *n* = 3). (**H**) ELISA assay showing eNAMPT concentrations in SN from M14/BiR and A375/BiR cells treated with 1µg/ml of BfA for 6 hours (*n* = 3).

Recent data indicate that melanoma cells actively release eNAMPT *in vitro* [21]. By using a commercially available sandwich ELISA assay, we confirmed variable levels of eNAMPT in conditioned media from 5 BRAF-wt and 7 BRAF-mutated (V600E) melanoma cell lines with no significant differences based on the presence of the BRAF (V600E) mutation (Figure 1A). No eNAMPT was found in unconditioned media added with 10% FCS (Figure 1A and Supplementary Figure 1A). *NAMPT* mRNA levels in these cell lines were directly correlated to the amount of eNAMPT (*r* = 0.85, *P* = 0.0004, Figure 1B). We also confirmed the presence of eNAMPT by western blot, analyzing conditioned media from 4 representative cell lines. Under reducing conditions, an anti-NAMPT-specific antibody highlighted a single band of ≈55 kDa corresponding to the monomer, while under non-reducing conditions NAMPT dimers/multimers (100–150 kDa) became visible (Figure 1C), in line with previous results [23].

Our recent data indicate that the acquisition of BRAF resistance in BRAF-mutated metastatic melanoma cells is accompanied by a significant increase of intracellular NAMPT (iNAMPT), which becomes the master regulator of NAD biosynthesis [22]. Here we asked whether high iNAMPT levels in BRAF-resistant (BiR) melanoma cells were paralleled by elevated extracellular concentrations of the enzyme. To the purpose, eNAMPT levels were compared in the tissue culture supernatants of A375 and M14 cells, in their sensitive (S) and BiR variants. eNAMPT levels in spent media from M14 cells rose from 5 ± 0.9 to 19.7 ± 2.5 ng/ml in S *vs* BiR variants. In A375/S cells eNAMPT levels were 0.65 ± 0.08 ng/ml, increasing to 6.1 ± 0.5 ng/ml in BiR cells (Figure 1D). These results are consistent with higher NAMPT mRNA levels and higher intracellular levels of NAD in M14 compared to A375 cells (Supplementary Figure 1B).

As a further validation of the ELISA assay, results were reproduced using Luminex technology, by exploiting a commercial assay for diabetes (Supplementary Figure 1C).

We then dosed eNAMPT in the plasma of immunocompromised mice xenografted with human A375/BiR cell lines by subcutaneous injection. Plasma was collected at the baseline and after tumor masses reached ≈1 mm^3^. As the antibodies used in the ELISA assay cross-react with the murine protein, a mean value of eNAMPT in normal mouse plasma of 6.6 ng/ml was measured. However, engraftment of human BiR melanoma cells induced a significant increase of eNAMPT levels (mean of 19.75 ng/ml, Figure 1E). These data are in line with a previous report showing that melanoma cells are responsible for the increase in eNAMPT levels in a mouse model [21]. Immunohistochemical staining performed on MIB1^+^ (i.e., proliferating) areas of the tumor confirmed high intracellular levels of NAMPT in live human xenografts in both BiR cell lines (Figure 1F).

### eNAMPT is constitutively released from melanoma cells

To rule out that eNAMPT in conditioned media was due to passive accumulation as a consequence of cell death, we monitored cellular viability alongside eNAMPT levels in BiR cell variants for 24–48–72 hours. In agreement with previous data [21, 23, 24], no significant modulation of apoptosis, as measured by annexin/propidium iodide FACS staining, could be highlighted, indicanting that eNAMPT release is an active process (Figure 1G). Even if the mechanisms behind eNAMPT secretion are incompletely understood, non-classical pathways seem to play a role [23, 24]. In line with these data, brefeldin A (BfA), an inhibitor of classical secretory pathways, failed to significantly modulate eNAMPT levels (Figure 1H).

### eNAMPT is abundant in plasma from BRAF-mutated MM patients and characterizes a set of patients with an unfavorable disease outcome

**Figure 2:**
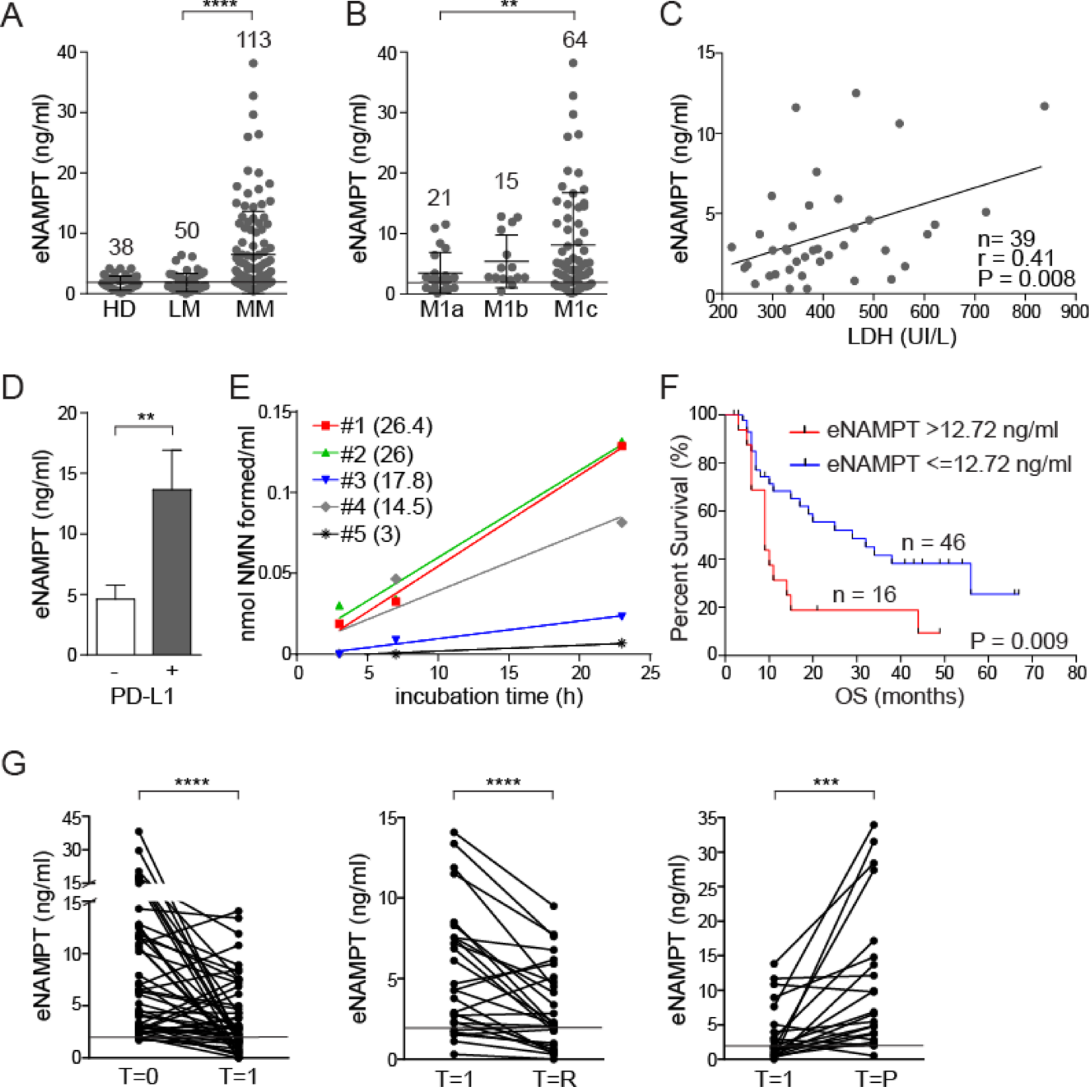
eNAMPT levels are significantly increased in patients with MM and correlate with response to therapy and overall survival (**A**) eNAMPT levels measured using an ELISA assay in sera from healthy donors (HD, *n* = 38), patients with a localized melanoma (LM, *n* = 50) or patients with metastatic disease (MM, *n* = 113). (**B**) ELISA assay showing eNAMPT concentrations in sera of stage IV melanoma patients subdivided in M1a, M1b and M1c subcategories. The horizontal line in (A–B) around 2 ng/ml indicated mean eNAMPT level in HD subjects. (**C**) Regression line showing a positive correlation between LDH (x-axis) and eNAMPT (y-axis) levels detected in 39 sera from MM patients. Pearson coefficient (r) and the corresponding *P* value are noted. (**D**) ELISA assay showing eNAMPT concentrations in sera from MM patients with PD-L1^+^ (*n* = 8) or PD-L1^-^ (*n* = 18) lesion (cut-off level >5% or <5% respectively). (**E**) eNAMPT activity determined in sera from 5 MM patients, with variable eNAMPT levels (indicated in brackets as ng/ml for each MM serum), as described in the Methods section. (**F**) Kaplan–Meyer curves showing overall survival (OS) of a cohort of 62 MM patients divided on the basis of eNAMPT levels (cut-off 12.72 ng/ml) at diagnosis. Log-rank test showed statistical significance. (**G**) ELISA assay showing eNAMPT concentrations in sera from 50 MM patients before and after therapy with a BRAFi alone or in combination with MEKi (T = 0). Graph on the left shows eNAMPT values before therapy and after 1 month of treatment (T = 1), where all patients showed some degree of response. The central panels shows eNAMPT levels in sera from 28 responsive MM patients at T = 1 (1 month of therapy) and at T = R (2–6 months later with consolidation of the clinical response). The graph on the right shows values in 22 progressive MM patients at T = 1 (1 month of therapy) and at T = P (at time of progression 6 months. The horizontal line, in all graphs indicates the mean eNAMPT level in HD subjects.

The above data provide the rationale to test eNAMPT concentrations in the plasma of BRAF-mutated MM patients. Results from the analysis of 163 patients at different disease stages, but always untreated, indicate that eNAMPT levels are significantly increased in patients with metastatic disease (MM, *n* = 113, mean eNAMPT levels 6.5 ± 0.7 ng/ml, *P* < 0.0001, Supplementary Table 1 and Figure 2A), compared to those in stages I-II localized melanoma (LM, *n* = 50, mean eNAMPT levels 1.9 ± 0.2 ng/ml, Figure 2A), who displayed eNAMPT concentrations comparable to healthy donors (HD, *n* = 38, mean eNAMPT levels 1.75 ± 0.2 ng/ml Figure 2A). Of note, eNAMPT levels were higher in males (*n* = 64, mean eNAMPT values 7.0 ± 1.0 ng/ml) compared to females (*n* = 49, mean eNAMPT values 5.5 ± 0.8 ng/ml). This difference, even if not statistically significant, was never highlighted in normal donors, including those examined for this work, and was likely due to a higher percentage of male patients in the M1c stage compared to females (70% males *vs* 55% females in M1c stage). In fact, when patients were stratified according to metastatic disease localization, it was apparent that patients in M1c stage [melanoma metastatic to any site with elevated lactate dehydrogenase (LDH) levels, or metastatic to visceral organs, excluding the lung] had significantly higher eNAMPT levels (mean 8.0 ± 1.1 ng/ml) than patients in M1a stage [skin or lymph node metastases with normal LDH, (mean 3.5 ± 0.7 ng/ml, *P* = 0.009)], while patients in M1b stage (metastasis to the lungs with normal LDH) showed intermediate values (mean 5.5 ± 1.1 ng/ml, Figure 2B). Consistently, a direct correlation between eNAMPT and LDH levels was highlighted in 39 patients for whom LDH measurement had been performed on the same sample used for eNAMPT testing (*r* = 0.41, *P* = 0.008, Figure 2C), in keeping with the notion that eNAMPT plasma levels correlate with tumor burden.

Moreover, for 26 patients we had data on the immunohistochemical expression of PD-L1, which is considered a negative prognostic marker [25]. Out of them, the 8 patients with a PD-L1^+^ lesion had significantly higher eNAMPT levels compared to negative ones (Figure 2D), suggesting a correlation between eNAMPT and an immunosuppressed microenvironment.

eNAMPT enzymatic activity in sera from 5 MM patients with variable eNAMPT levels was then measured using an *ad hoc* devised assay [26]. Both substrates [nicotinamide and phosphoribosyl pyrophosphate (PRPP)], as well as ATP, which is known to increase the catalytic efficiency of the enzyme [27], were added to the reaction mixtures. Under these conditions, 4/5 sera showed formation of nicotinamide mononucleotide (NMN), the product of the NAMPT-catalyzed reaction (mean activity of 3.7 ± 0.9 nmol NMN/ml). Interestingly, the plasma where NAMPT activity could not be measured was the one with the lowest eNAMPT levels (Figure 2E).

Follow up data were available for 62 MM patients. By applying recursive partitioning analysis to this cohort an eNAMPT cut-off of 12.72 ng/ml was determined. The 16 patients with eNAMPT levels >12.72 ng/ml showed inferior overall survival (OS, median 9 months), compared to the 46 patients with eNAMPT levels ≤12.72 ng/ml (median 29 months; log-rank test *P* = 0.009, Figure 2F). All patients (16/16) with high levels of eNAMPT experienced disease progression, at variance with those with low levels of eNAMPT where progression occurred only in 11/46 (24%).

### eNAMPT levels decrease in response to therapy, increasing again upon disease progression

Plasmatic NAMPT levels were then prospectically followed at different time points in a cohort of 62 stage IV melanoma patients treated with BRAFi alone or in combination with MEK inhibitors (MEKi). A sample from each patient was obtained at the baseline, after 4 weeks of therapy and at a third time point varying between 2 and 6 months. We excluded from this analysis patients for whom eNAMPT levels fell within the normal range, by applying a cut-off of ≥1.75 ng/ml. In the remaining 50 patients, a significant drop in eNAMPT levels was observed at the first time point in the analysis, where all patients experienced some level of response to therapy (mean eNAMPT levels pre-therapy [T = 0] 8.4 ± 0.9 ng/ml, mean eNAMPT after 1 month [T = 1] of therapy 4 ± 0.5 ng/ml, *P* < 0.0001, Figure 2G). eNAMPT levels were then analyzed at a later time point (varying from 2–6 months after beginning of therapy), where patients had either consolidated the clinical response or were experiencing progression. In the patients that consolidated the response (T = R, *n* = 28), eNAMPT was further decreased (mean 3.2 ± 0.5 ng/ml, *P* = 0.0001, Figure 2G), while it increased in the 19/22 patients with disease progression (T = P, mean 11.1 ± 2.2 ng/ml, *P* = 0.0003, Figure 2G).

## DISCUSSION

This work focuses on the analysis of eNAMPT levels in patients with BRAF-mutated melanoma. NAMPT is an intracellular enzyme that controls a critical step in one of the pathways leading to NAD biosynthesis. It has long been known that NAMPT is present in extracellular fluids, where it was originally described as a cytokine-like molecule, which promoted growth and differentiation of B cell precursors [9]. We now know that eNAMPT is elevated in conditions of chronic inflammation, including cancer [8, 16], where it is likely to contribute to local immunosuppression [19].

The choice of melanoma derives from our recent study, which indicates that NAMPT becomes the dominant NAD-biosynthetic enzyme in BRAF-resistant melanoma cell lines and patients BRAF-mutated [22]. Here we show that melanoma cells can release eNAMPT, most likely through a non-classical secretory pathway, and independently of their BRAF mutational status, suggesting that these cells are intrinsically able to secrete this enzyme. Accordingly, eNAMPT is found in the plasma of mice xenografted with human melanoma cells. An important finding is that spent media from BRAF-mutated cells that were rendered resistant to BRAFi and that overexpress iNAMPT, contain significantly higher eNAMPT levels compared to the sensitive counterparts, suggesting that the extracellular version of the molecule is proportional to the intracellular one.

We then evaluated eNAMPT in 163 patients with BRAF-mutated melanoma. The first indication here is that eNAMPT levels increase substantially when the disease becomes metastatic, while patients with a localized disease were undistinguishable from healthy controls. Second, the amount of eNAMPT shows a direct correlation with LDH, which is considered a marker of tumor burden. Third, the highest levels of eNAMPT are typical of patients with visceral metastases (M1c), compared to patients in the M1a stage. A subgroup of 62 of these patients was treated with BRAFi or MEKi, with follow-up data available for analysis. The first observation is that patients with eNAMPT levels above the threshold of 12.72 ng/ml, defined based on a recursive partitioning analysis, died significantly earlier compared to patients with lower eNAMPT levels. Importantly, 16/16 of patients with >12.72 ng/ml of eNAMPT experienced disease progression, at variance with only 11/46 in the subgroup with lower eNAMPT levels. Furthermore, eNAMPT levels in treated patients could be followed during the course of therapy. Results show that 1 month after beginning therapy with BRAFi/MEKi, when most patients were responding, eNAMPT levels dropped significantly, but picked up again specifically in patients who relapsed. On the contrary, they decreased further over the course of 2–6 months in patients that consolidated responses to targeted therapies.

Overall, these data suggest that eNAMPT should be further studied as a disease marker for melanoma patients with a BRAF-mutated tumor. From the functional stand point, eNAMPT may exert more complex functions in the melanoma microenvironment than being a mere reflection of the intracellular levels of the enzyme. Grolla *et al.* recently demonstrated that eNAMPT released by melanoma cell line *in vitro* had paracrine and autocrine effects activating nuclear factor kappa B (NF-KB) signaling and increasing colony-formation in anchorage-independent conditions [21]. On the other side, nothing is known yet on the effects of eNAMPT in the tumor microenvironment. However, it is of interest to note that both melanoma cells and tumor-infiltrating macrophages can express TLR4 [28–30], which was recently identified as the eNAMPT receptor [20]. A tumor-supportive, immune-suppressive function for eNAMPT was shown in chronic lymphocytic leukemia [19] and in other tumor models [8]. It is however also possible that the biological actions of eNAMPT also depend on its enzymatic activity. Our data indicate that the extracellular version of the molecule is enzymatically active, provided that the substrates are available. If so, eNAMPT could also lead to the modulation of extracellular NAD concentrations, likely affecting purinergic receptor signaling [31].

In conclusion, this work shows that eNAMPT levels increase during melanoma progression and upon BRAFi resistance development, negatively correlating with patient overall survival. Further studies are needed to confirm that eNAMPT is a marker of disease burden and possibly an independent negative prognostic factor in BRAF-mutated MM patients, as well as a critical element in the induction of immunosuppressive and tumor-promoting conditions in the melanoma microenvironment.

## MATERIALS AND METHODS

### Patients

All patients enrolled in the study provided written informed consent. BRAF (V600E) mutational status was determined by Sanger sequencing. Patient plasma or sera were collected in accordance with the Institutional Review Board and the Declaration of Helsinki. All patients received BRAFi alone (Dabrafenib or Vemurafenib) or a combination of BRAFi and MEKi (Trametinib). Clinical features of the metastatic patients are indicated in Supplementary Table 1. For some patients information on the levels of LDH (UI/mL) or PD-L1 expression on tissue biopsies [32] were available.

Blood samples from healthy donors were obtained through the local blood bank.

### Cell lines

BRAF WT cell lines were: SK-MEL-2, 501-MEL, 4778D-MEL WKMEL and 197-MEL. BRAF-mutated (V600E) cell lines were: A375, M14, SK-MEL-5, COLO38, FO-1, 1061-MEL, MEL-MAI. BRAF mutational status (V600E) was confirmed by Sanger sequencing. A375 and M14 BRAF-resistant (BiR) cells were generated by adding increasing concentrations of the BRAF inhibitor (BRAFi) Dabrafenib (GlaxoSmithKline) to complete medium, reaching the final concentration of 1.6 µM in ≈10 weeks. BiR cells were then maintained under these conditions. Culture conditions are described in Supplementary Material and Methods (SMM).

### Xenograft models

A375 and M14 BiR melanoma cells (5 × 10^6^) were resuspended in Matrigel^®^ (Corning) and injected into the right and left flank of 6–8-week old male NOD/SCID/gamma chain^-/-^(NSG) mice (Charles River). Once tumors became palpable mice were sacrificed and tumors processed. Plasma samples were obtained at the baseline and at sacrifice for A375 BiR engraftments.

### Assays to quantify eNAMPT in biological fluids

eNAMPT concentrations in plasma and culture supernatants were determined using human NAMPT Enzyme-Linked Immunosorbent Assay (ELISA) kit (Adipogen). To validate results, some samples were analyzed also through Luminex Technology using Bio-Plex Pro Human Diabetes Assay panel (Bio-Rad) that includes NAMPT.

### Preparation of culture supernatants and Western blot

For eNAMPT measurement melanoma cells (1.5x10^5^) were seeded in 24-well plates in media plus 0.1%FCS (Sigma) for 24 hours. Spent media was then collected and concentrated 10x using 30 kDa cut off filters (Amicon Ultra centrifugal Filters, Millipore) and then analyzed by western blot as described in SMM.

### Treatments with Brefeldin A

M14 and A375 BiR (2.5 × 10^5^) were seeded in 24-well plates in complete medium and treated or not with Brefeldin A (BfA, 1 µg/ml, Sigma) for 6 hours before collecting supernatants and performing the ELISA assay.

### NAMPT activity in plasma

NAMPT activity was determined by a multicoupled fluorometric assay, developed to measure NAMPT activity in cell crude extracts and biological fluids [26].

### RNA extraction and quantitative real-time polymerase chain reaction (qRT-PCR)

RNA was extracted and RT-PCR performed as described in SMM.

### Cell viability

Cell viability was measured using the Annexin-V-FITC Apoptosis Kit (Valter Occhiena). Data were acquired using a FACSCanto II cytofluorimeter and processed with DIVA version 8 (BD Biosciences).

### Immunohistochemistry

Formalin-fixed, paraffin-embedded skin sections were stained as described in SMM. Images were acquired using a CANON EOS 600D camera fitted to AXIO Lab A1 Zeiss microscope.

### Statistical analyses

Mann–Whitney U (unpaired data), or Wilcoxon signed rank (paired data) tests were used to compare continuous variables. Overall survival (OS) was calculated as the time from melanoma diagnosis to death. Patients who neither recurred nor died at the end of the study were censored at the time of the last follow-up. Survival curves were estimated with the Kaplan-Meier method and compared by means of Log-rank test. Recursive partitioning analysis (RPA) was applied to define the best eNAMPT cut-off in our series. ^*^P ≤ 0.05, ^**^P ≤ 0.01, ^***^P ≤ 0.001, ^****^P ≤ 0.0001.

Statistical analyses were performed with GraphPad version 6.0, SAS version 9.4 and R version 3.2.5 softwares.

## SUPPLEMENTARY MATERIALS FIGURE AND TABLE



## References

[1] Chiarugi A, Dolle C, Felici R, Ziegler M (2012). The NAD metabolome—a key determinant of cancer cell biology. Nature reviews Cancer.

[2] Imai S, Guarente L (2014). NAD+ and sirtuins in aging and disease. Trends Cell Biol.

[3] Magni G, Orsomando G, Raffelli N, Ruggieri S (2008). Enzymology of mammalian NAD metabolism in health and disease. Front Biosci.

[4] Grahnert A, Klein C, Schilling E, Wehrhahn J, Hauschildt S (2011). Review: NAD + : A modulator of immune functions. Innate Immun.

[5] Houtkooper RH, Canto C, Wanders RJ, Auwerx J (2010). The secret life of NAD+: an old metabolite controlling new metabolic signaling pathways. Endocr Rev.

[6] Imai S, Yoshino J (2013). The importance of NAMPT/NAD/SIRT1 in the systemic regulation of metabolism and ageing. Diabetes Obes Metab.

[7] Ruggieri S, Orsomando G, Sorci L, Raffaelli N (2015). Regulation of NAD biosynthetic enzymes modulates NAD-sensing processes to shape mammalian cell physiology under varying biological cues. Biochim Biophys Acta.

[8] Garten A, Schuster S, Penke M, Gorski T, de Giorgis T, Kiess W (2015). Physiological and pathophysiological roles of NAMPT and NAD metabolism. Nat Rev Endocrinol.

[9] Samal B, Sun Y, Stearns G, Xie C, Suggs S, McNiece I (1994). Cloning and characterization of the cDNA encoding a novel human pre-B-cell colony-enhancing factor. Molecular and cellular biology.

[10] Sun Z, Lei H, Zhang Z (2013). Pre-B cell colony enhancing factor (PBEF), a cytokine with multiple physiological functions. Cytokine Growth Factor Rev.

[11] Moschen AR, Gerner RR, Tilg H (2010). Pre-B cell colony enhancing factor/NAMPT/visfatin in inflammation and obesity-related disorders. Curr Pharm Des.

[12] Jia SH, Li Y, Parodo J, Kapus A, Fan L, Rotstein OD, Marshall JC (2004). Pre-B cell colony-enhancing factor inhibits neutrophil apoptosis in experimental inflammation and clinical sepsis. J Clin Invest.

[13] Garten A, Petzold S, Schuster S, Korner A, Kratzsch J, Kiess W (2011). Nampt and its potential role in inflammation and type 2 diabetes. Handb Exp Pharmacol.

[14] Gesing J, Scheuermann K, Wagner IV, Loffler D, Friebe D, Kiess W, Schuster V, Korner A (2017). NAMPT serum levels are selectively elevated in acute infectious disease and in acute relapse of chronic inflammatory diseases in children. PLoS One.

[15] Shackelford RE, Mayhall K, Maxwell NM, Kandil E, Coppola D (2013). Nicotinamide phosphoribosyltransferase in malignancy: a review. Genes Cancer.

[16] Grolla AA, Travelli C, Genazzani AA, Sethi JK (2016). Extracellular nicotinamide phosphoribosyltransferase, a new cancer metabokine. Br J Pharmacol.

[17] Sampath D, Zabka TS, Misner DL, O’Brien T, Dragovich PS (2015). Inhibition of nicotinamide phosphoribosyltransferase (NAMPT) as a therapeutic strategy in cancer. Pharmacology & therapeutics.

[18] Galli U, Travelli C, Massarotti A, Fakhfouri G, Rahimian R, Tron GC, Genazzani AA (2013). Medicinal Chemistry of Nicotinamide Phosphoribosyltransferase (NAMPT) Inhibitors. J Med Chem.

[19] Audrito V, Serra S, Brusa D, Mazzola F, Arruga F, Vaisitti T, Coscia M, Maffei R, Rossi D, Wang T, Inghirami G, Rizzi M, Gaidano G (2015). Extracellular nicotinamide phosphoribosyltransferase (NAMPT) promotes M2 macrophage polarization in chronic lymphocytic leukemia. Blood.

[20] Camp SM, Ceco E, Evenoski CL, Danilov SM, Zhou T, Chiang ET, Moreno-Vinasco L, Mapes B, Zhao J, Gursoy G, Brown ME, Adyshev DM, Siddiqui SS (2015). Unique Toll-Like Receptor 4 Activation by NAMPT/PBEF Induces NFkappaB Signaling and Inflammatory Lung Injury. Sci Rep.

[21] Grolla AA, Torretta S, Gnemmi I, Amoruso A, Orsomando G, Gatti M, Caldarelli A, Lim D, Penengo L, Brunelleschi S, Genazzani AA, Travelli C (2015). Nicotinamide phosphoribosyltransferase (NAMPT/PBEF/visfatin) is a tumoural cytokine released from melanoma. Pigment Cell Melanoma Res.

[22] Audrito V, Managò A, La Vecchia S, Zamporlini F, Vitale N, Baroni G, Cignetto S, Serra S, Bologna C, Stingi A, Arruga F, Vaisitti T, Massi D (2018). Nicotinamide phosphoribosyltransferase (NAMPT) as a Therapeutic Target in BRAF-mutated metastatic melanoma. J Natl Cancer Inst.

[23] Garten A, Petzold S, Barnikol-Oettler A, Korner A, Thasler WE, Kratzsch J, Kiess W, Gebhardt R (2010). Nicotinamide phosphoribosyltransferase (NAMPT/PBEF/visfatin) is constitutively released from human hepatocytes. Biochem Biophys Res Commun.

[24] Revollo JR, Korner A, Mills KF, Satoh A, Wang T, Garten A, Dasgupta B, Sasaki Y, Wolberger C, Townsend RR, Milbrandt J, Kiess W, Imai S (2007). Nampt/PBEF/Visfatin regulates insulin secretion in beta cells as a systemic NAD biosynthetic enzyme. Cell Metab.

[25] Audrito V, Serra S, Stingi A, Orso F, Gaudino F, Bologna C, Neri F, Garaffo G, Nassini R, Baroni G, Rulli E, Massi D, Oliviero S (2017). PD-L1 up-regulation in melanoma increases disease aggressiveness and is mediated through miR-17-5p. Oncotarget.

[26] Zamporlini F, Ruggieri S, Mazzola F, Amici A, Orsomando G, Raffaelli N (2014). Novel assay for simultaneous measurement of pyridine mononucleotides synthesizing activities allows dissection of the NAD(+) biosynthetic machinery in mammalian cells. FEBS J.

[27] Burgos ES, Schramm VL (2008). Weak coupling of ATP hydrolysis to the chemical equilibrium of human nicotinamide phosphoribosyltransferase. Biochemistry.

[28] Goto Y, Arigami T, Kitago M, Nguyen SL, Narita N, Ferrone S, Morton DL, Irie RF, Hoon DS (2008). Activation of Toll-like receptors 2, 3, and 4 on human melanoma cells induces inflammatory factors. Mol Cancer Ther.

[29] Zhang QQ, Zhou DL, Ding Y, Liu HY, Lei Y, Fang HY, Gu QL, He XD, Qi CL, Yang Y, Lan T, Li JC, Gong P (2014). Andrographolide inhibits melanoma tumor growth by inactivating the TLR4/NF-kappaB signaling pathway. Melanoma Res.

[30] Zarember KA, Godowski PJ (2002). Tissue expression of human Toll-like receptors and differential regulation of Toll-like receptor mRNAs in leukocytes in response to microbes, their products, and cytokines. J Immunol.

[31] Chini CCS, Tarrago MG, Chini EN (2017). NAD and the aging process: Role in life, death and everything in between. Mol Cell Endocrinol.

[32] Massi D, Brusa D, Merelli B, Falcone C, Xue G, Carobbio A, Nassini R, Baroni G, Tamborini E, Cattaneo L, Audrito V, Deaglio S, Mandala M (2015). The status of PD-L1 and tumor-infiltrating immune cells predict resistance and poor prognosis in BRAFi-treated melanoma patients harboring mutant BRAFV600. Ann Oncol.

