# Supplementary material for: Extracellular nicotinamide phosphoribosyltransferase (eNAMPT) is a novel marker for patients with BRAF-mutated metastatic melanoma
Source: Oncotarget. 2018 Apr 10;9(27):18997–9005. doi: 10.18632/oncotarget.24871 (PMC5922372; doi:10.18632/oncotarget.24871)
Supplement: Supplementary file 1 [file oncotarget-09-18997-s001.pdf]

Extracellular nicotinamide phosphoribosyltransferase (eNAMPT) is a novel marker for patients with BRAF-mutated metastatic melanoma

SUPPLEMENTARY MATERIALS

MATERIALS AND METHODS

Cell lines culture

Cell lines were cultured in RPMI-1640 with 10% of fetal calf serum (FCS) and 10 IU/ml of penicillin/streptomycin (all from Sigma), referred to as complete medium). Only MEL-197 were cultured in Dulbecco's modified eagle's medium (DMEM, Thermo Fisher).

Western blot

Conditioned media derived from indicated cell lines cultured at the same conditions (in reducing or non-reducing conditions) were resolved by sodium dodecyl sulfate– polyacrylamide gel electrophoresis and transferred to nitrocellulose membranes (Bio-Rad). A recombinant form of NAMPT (rec) was prepared in-house [1] and used as control. Anti-NAMPT monoclonal antibody from Bethyl Laboratories, while the goat anti-rabbit HRP- conjugated secondary antibody was from Santa Cruz Biotechnology. Images were acquired using the ImageQuant Las4000 gel imager (GE Healthcare).

RNA extraction and quantitative real-time polymerase chain reaction (qRT-PCR)

RNA was extracted using RNeasy Plus Mini kit (Qiagen) and converted to complementary DNA using the High Capacity cDNA Reverse Transcription kit (Thermo Fisher). qRT-PCR was performed using the 7900 HT Fast Real Time PCR system (SDS2.3 software) using the TaqMan assays (Thermo Fisher) Hs00237184_m1 (NAMPT). Actin was used as housekeeping gene: Hs99999903_m1 (ACTB). Relative gene expression on was calculated as described [1].

Immunohistochemistry

Immunohistochemistry was carried out on 4 µm-thick sections cut from formalin-fixed, paraffin-embedded tissue samples and immunostained according to standard procedures. Briefly, antigen retrieval was performed by immersing the slides in a thermostat bath containing Epitope Retrieval Solution EDTA (pH 9.0; 20 minutes at 98° C, Dako), followed by cooling (20 minutes, room temperature). Section staining was automatically performed using Ventana BenchMark ULTRA Immunostainer (Ventana Medical SystemsThe Ventana staining procedure included pretreatment with cell conditioner 1, followed by incubation with the antibody. For all antibodies, the signals were developed with Universal Alkaline Phosphatase Red Detection Kit (all reagents from Ventana). Nuclei were counterstained with Mayer hematoxylin. Negative control was performed by substituting the primary antibody with a mouse-rabbit serum (Dako). Control sections were treated in parallel with the samples.

Primary antibodies used for immunohistochemistry were: anti-NAMPT (clone OMNI379, 25A-0025, Adipogen), anti-Ki-67 (clone 30-9 MIB1, ready to use, Ventana).

REFERENCES

1. Audrito V, Serra S, Brusa D, Mazzola F, Arruga F, Vaisitti T, Coscia M, Maffei R, Rossi D, Wang T, Inghirami G, Rizzi M, Gaidano G, et al. Extracellular nicotinamide phosphoribosyltransferase (NAMPT) promotes M2 macrophage polarization in chronic lymphocytic leukemia. Blood. 2015; 125:111–123.

Supplementary Table 1: Main clinical characteristics of the cohort of BRAF-mutated metastatic melanoma (MM) patients studied

Variable	No. (%)
BRAF V600E MM patients	113
Median age at diagnosis of MM disease (years)	56
Sex (male)	64 (56.6)
Sex (female)	49 (43.4)
Stage classification	
M1a	22 (21.6)
M1b	15 (14.7)
M1c	65 (63.7)

Table summarizing clinical features of the cohort of 113 patients with metastatic melanoma (stage IV) carrying BRAFV600E mutation analyzed in the study.

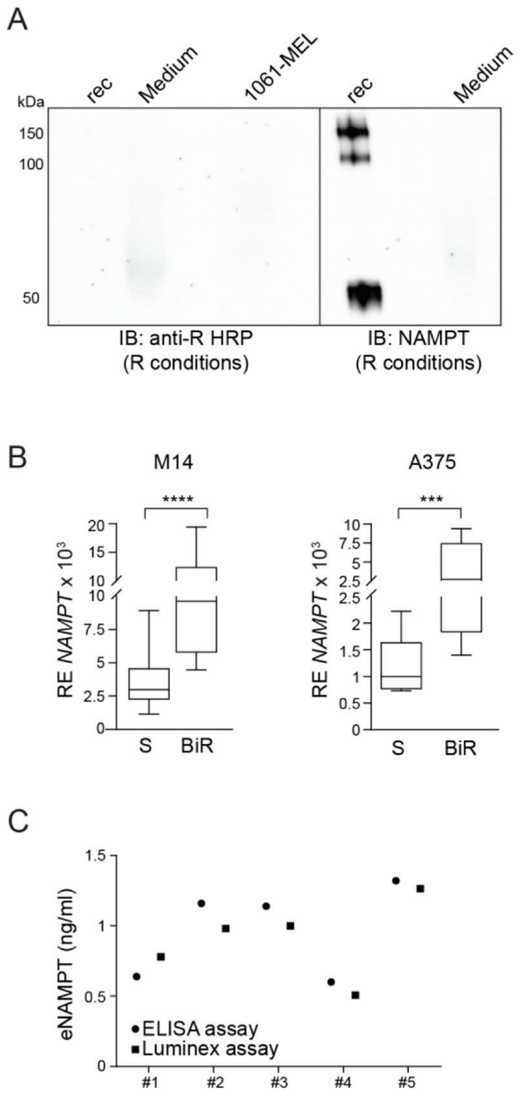

Supplementary Figure 1: NAMPT in melanoma cells. (**A**) Western blot showing not cross-reactivity of secondary antibody [anti-rabbit (R) HRP] on medium (RPMI + 10% FCS) or supernatant from 1061-MEL cell line samples (left panel). Right panel of western blot shows specific signal for NAMPT only in rec (recombinant NAMPT protein) but not only in medium. All samples were run in reducing conditions. (**B**) Box plots reporting NAMPT mRNA expression levels in S and BiR cells for M14 and A375 cell line variants. (**C**) Graph reporting eNAMPT values in 5 different supernatants samples measured using ELISA Adipogen assay (dots) and Luminex method (squares).
